# The Bayesian audit: evaluating the proportionality of scientific claims to evidence — a case study on social priming and walking speed

**DOI:** 10.3389/fpsyg.2026.1799078

**Published:** 2026-05-22

**Authors:** Tommaso Costa

**Affiliations:** Department of Psychology, University of Turin, Turin, Italy

**Keywords:** Bayes factor, Bayesian inference, prior assumptions, replication crisis, scientific claims

## Abstract

Across psychology, bold empirical claims often outpace the evidential support on which they rest. The replication crisis has shown that statistical significance alone provides little guidance about what should rationally be believed. To address this gap between results and rhetoric, we introduce the Bayesian audit—a conceptual and normative framework, rather than a new statistical method, for evaluating whether scientific claims are proportionate to the strength of their evidence. The audit proceeds by identifying the claim, specifying priors, translating the empirical evidence into a likelihood-based measure, updating to obtain posterior belief, testing sensitivity, and synthesizing proportional conclusions. Applied to a well-known case in social psychology—the “elderly priming” study by Bargh et al. (1996)—the audit reveals that the original finding corresponds to only modest Bayesian evidence (Bayes factor ≈ 3). Under reasonable priors (0.05–0.20), the posterior probability that the effect is genuine remains below 0.5, and replication attempts provide limited additional evidential impact at the level of individual studies. The exercise illustrates how strong theoretical language can emerge from weak evidential shifts and how Bayesian reasoning can realign scientific communication with inferential logic. The framework is particularly relevant for psychological science, where replication failures often reflect over-claiming rather than data absence, and where proportional reasoning can help restore coherence between evidence and belief.

## Introduction

Across the behavioral sciences, striking empirical claims often travel farther than the evidential scaffolding that originally supported them. The replication crisis has made this tension explicit: when study power is low, biases are present, and the base rate of true effects is modest, many published findings will overstate what the data can rationally support (Ioannidis, 2005; Open Science Collaboration, 2015). Following common usage, we distinguish between reproducibility—the ability to obtain the same results using the same data and analysis—and replicability, the ability to obtain consistent results across independent studies. The present work is primarily concerned with the latter, as mismatches between claims and evidence typically emerge when findings fail to replicate. The result is an evidential–rhetorical gap between what is claimed and what is warranted. A methodological response must therefore assess not just statistical significance, but the proportionality of scientific claims to the strength of the evidence that sustains them (Jaynes, 2003; Wagenmakers et al., 2018).

The classic social priming experiment by Bargh et al. (1996), in which participants subliminally exposed to words related to old age walked more slowly afterward, encapsulates this problem. The claim that unconscious semantic activation can directly alter overt motor behavior is powerful and theoretically seductive. Yet, subsequent replications (Doyen et al., 2012; Harris et al., 2013) failed to reproduce the original finding or found that the effect depended on experimenter expectations. The episode vividly illustrates how surprising claims, supported by small samples and marginal *p*-values, can gain disproportionate scientific and cultural visibility (Yong, 2012; Kahneman, 2017).

A principled remedy is to reframe the evaluation of empirical results in explicitly Bayesian terms. Rather than asking whether data clear an arbitrary threshold, we can ask how strongly those data should shift rational belief, conditional on prior plausibility and model assumptions (Good, 1950; Jeffreys, 1961; Jaynes, 2003). Work on Bayes factors has long emphasized that *p*-values are not measures of evidential strength and that modest results often correspond to only anecdotal evidence (Wagenmakers et al., 2010; Etz and Vandekerckhove, 2016). Complementary approaches such as the reverse-Bayes method explicitly ask what prior would be required for a reported result to be credible, directly quantifying the proportionality between claim and evidence (Matthews, 2018; Held, 2020; Held and Ott, 2018).

In this article, we formalize a general and transparent procedure for conducting such evaluations: the Bayesian audit. The audit is a six-step, claim-centered framework designed to assess whether a scientific assertion is epistemically proportional to the evidence on which it rests. It involves identifying the strong or general claim, making priors explicit and justifiable, translating reported data into a likelihood (for example, Bayes factor, diagnostic accuracy, or effect-size model), updating the prior to obtain a posterior belief, performing sensitivity analyses across plausible priors, and providing a proportional synthesis of evidential strength. We illustrate this framework using the social priming case, showing how a seemingly decisive claim reduces to modest posterior support once prior plausibility and realistic uncertainty are accounted for. More broadly, we argue that systematic Bayesian audits can help align scientific rhetoric with inferential logic, bridging the gap between what is statistically detected and what is rationally believed.

Throughout the article, we distinguish between evidence—quantified by the Bayes factor as the extent to which data shift rational belief—and belief, represented by posterior probabilities that combine evidence with prior plausibility.

## Related work

The methodological critique of statistical inference in psychology has a long history, tracing back to the recognition that null-hypothesis significance testing (NHST) conflates statistical detection with epistemic confirmation. As early as the mid-twentieth century, authors such as Good (1950), Jeffreys (1961), and Lindley (1957) proposed Bayesian inference as a coherent alternative to frequentist hypothesis testing, emphasizing that evidence should be expressed as a change in rational belief rather than as a binary decision rule. Jaynes (2003) later unified these ideas under the broader view of probability as extended logic, arguing that scientific reasoning itself can be formalized as a sequence of probabilistic updates.

The renewed interest in Bayesian reasoning within psychology has been largely driven by the reproducibility crisis and by the limitations of *p*-values as indicators of evidential strength. Wagenmakers et al. (2010) and Rouder et al. (2009) popularized the use of Bayes factors in experimental psychology, showing how they quantify relative evidence for competing models without relying on arbitrary thresholds. Subsequent developments made Bayesian analysis accessible through software such as JASP (Love et al., 2019; Morey and Rouder, 2022), leading to widespread adoption in cognitive and social research. These contributions collectively reframed inference from dichotomous significance testing toward a graded evaluation of evidential strength (Etz and Vandekerckhove, 2016; Kruschke and Liddell, 2018).

Parallel to this movement, several authors proposed methods for reassessing published findings through Bayesian re-analysis. The “reverse-Bayes” approach (Matthews, 2018; Held and Ott, 2018; Held, 2020) inverts the usual direction of inference by asking what prior distribution would be required to render a reported *p*-value or confidence interval credible. This perspective has proven useful in quantifying the tension between observed results and their prior plausibility, especially in fields prone to publication bias or underpowered studies. Related efforts such as the credibility analysis of Held (2020) and the skeptical priors framework of Dienes (2014, 2016) converge on the idea that scientific claims should be evaluated in proportion to prior belief and data strength rather than by convention alone.

More broadly, Bayesian reassessment techniques have been applied to meta-analyses, replication studies, and controversial empirical effects (van Ravenzwaaij and Wagenmakers, 2019; Verhagen and Wagenmakers, 2014). These approaches typically operate at the level of evidence quantification, yet they do not provide a systematic procedure for auditing the coherence between the *claim* as stated in the literature and the *evidence* that supports it. The concept of a “Bayesian audit” proposed here builds directly on this foundation: it extends Bayesian re-analysis from data evaluation to claim evaluation, treating scientific assertions themselves as hypotheses whose evidential proportionality can be quantified. In doing so, it bridges the logic of Bayesian inference with the epistemic demands of reproducibility and transparent reasoning (Tables 1, 2).

**Table 1:** Conceptual and operational differences among reverse-Bayes, credibility analysis, and Bayesian audit.

Approach	Core question	Operational focus	Output	Primary reference
Reverse-Bayes	“What prior would make the observed result credible?”	Inverts the inference: computes the prior required for a given p-value or CI to yield a target posterior.	Implied prior probability or Bayes factor consistent with observed data.	Matthews (2018) and Held and Ott (2018)
Credibility analysis	“Is the reported result credible under a specified skeptical prior?”	Tests whether the posterior excludes zero (or null) given a predefined skeptical prior.	Credibility ratio or adjusted credibility interval.	Held (2020)
Bayesian audit	“Is the scientific *claim* proportional to its evidential support?”	Six-step procedure: identify claim, set priors, derive likelihood, update posterior, test sensitivity, and synthesize proportional conclusion.	Posterior belief and qualitative proportional assessment linking data strength to claim language.	Present work

**Table 2:** Posterior probabilities for several prior assumptions.

Prior probability (*p*_0_)	Prior odds	BF_10_	Posterior odds	Posterior probability (p_post)
0.20	0.25	3	0.75	0.43
0.10	0.111	3	0.333	0.25
0.05	0.0526	3	0.1578	0.14

## Comparison with related Bayesian approaches

Several existing Bayesian approaches also aim to reassess the strength or credibility of published findings, most notably the *reverse-Bayes* method (Matthews, 2018; Held and Ott, 2018) and *credibility analysis* (Held, 2020). These methods are closely aligned with the rationale of the Bayesian audit, but they differ in focus and operational scope.

The reverse-Bayes approach inverts inference to determine the prior necessary to render a given result credible. Credibility analysis quantifies whether a reported result remains credible under a specified skeptical prior.

The Bayesian audit, by contrast, is designed as a broader procedural framework that integrates these quantitative tools within an explicit epistemic workflow centered on *claims* rather than on isolated statistical results. It links priors, likelihoods, and posterior belief directly to the proportionality of the scientific language used in the original report.

## The Bayesian audit: a six-step framework

The Bayesian audit is a conceptual framework designed to assess whether the strength of a scientific claim is proportionate to the evidence that supports it. It combines the logic of Bayesian updating (Jaynes, 2003) with the practical need to evaluate published results in context. The framework consists of six steps: identifying the claim, specifying priors, translating the evidence into a likelihood-based measure, updating to posterior belief, assessing sensitivity, and synthesizing proportional conclusions.

The present work is illustrative rather than empirical: the social priming case study serves solely to demonstrate how the framework operates in practice, using previously published results without introducing new data or meta-analytic aggregation.

### Step 1: Identify the claim

The first step is to extract from a study the precise claim that is being made. Scientific reports often mix empirical findings, theoretical interpretations, and rhetorical generalizations. The audit begins by isolating the assertive content—the statement whose truth is being implicitly asserted, typically causal (“A causes B”) or general (“X predicts Y across contexts”). The strength of the claim determines the level of prior plausibility it requires. As Goodman (1999) and Gelman and Carlin (2014) have argued, overstating what a statistical result implies is a key source of irreproducibility.

### Step 2: Specify the priors

The second step is to make explicit the prior probability that the claim is true before seeing the data. Priors may be informed by theory, previous literature, expert consensus, or base rates of true effects in similar domains (Matthews, 2018; Held, 2020). In diagnostic contexts, this corresponds to the pre-test probability. The goal is not to find a “true” prior, but to make background assumptions transparent and open to scrutiny.

Prior odds are defined as:

$$\mathrm{Odd}s_{\left(\text{prior}\right)}=\frac{p_{0}}{\left(1-p_{0}\right)}$$

In practice, priors may be informed by theoretical expectations, empirical base rates, or expert judgment. The values considered in the present analysis are illustrative rather than formally elicited. In the absence of a standardized procedure for prior elicitation, the audit proceeds by examining a range of plausible assumptions, with the aim of making explicit how conclusions depend on prior beliefs rather than identifying a single “correct” prior. The aim is not to identify a single correct prior, but to make underlying assumptions explicit and open to critical evaluation.

### Step 3: Translate empirical evidence into a likelihood-based measure of evidence

The third step involves translating the empirical evidence into a likelihood-based measure of evidence, summarizing how probable the observed data are under the hypothesis versus its alternative. Depending on the nature of the study, this can take the form of a Bayes factor (Jeffreys, 1961; Wagenmakers et al., 2010), a measure of diagnostic performance (sensitivity and specificity), or another suitable summary statistic.

The Bayes factor expresses the relative support provided by the data:

$$BF_{10}=\frac{P(D|H_{1})}{P(D|H_{0})}$$

where *D* denotes the data, *H*_1_ the hypothesis (claim true), and *H*_0_ the null.

Bayes factors can be obtained analytically or approximated from reported test statistics using established methods, such as those proposed by Rouder et al. (2009) or Held and Ott (2018).

### Step 4: Update to obtain the posterior

Once the likelihood is obtained, the posterior odds are calculated as the product of the prior odds and the Bayes factor:

$$\mathrm{Odd}s_{\left(\text{post}\right)}=BF_{10}\times \mathrm{Odd}s_{\left(\text{prior}\right)}$$

The posterior probability that the claim is true follows directly as:

$$p_{\left(\text{post}\right)}=\frac{\mathrm{Odd}\;}{\left(1+\mathrm{Odd}s_{\left(\text{post}\right)}\right)}$$

This value quantifies the degree of belief that remains after combining prior information with empirical evidence. It is analogous to the positive predictive value in diagnostic reasoning, widely used in medical statistics (Gigerenzer and Hoffrage, 1995).

### Step 5: Assess sensitivity

Because priors are inevitably uncertain, sensitivity analyses can target either prior model probabilities or prior distributions on parameters. In the present audit, the focus is deliberately placed on prior model probabilities, as the goal is to assess the proportionality of claims rather than the robustness of a specific effect-size model. Sensitivity analysis can be conducted by varying *p*_0_ or *BF*_10_ across reasonable ranges and observing how $p_{\text{post}}$ changes. A claim that is only supported under a narrow or extreme set of priors lacks epistemic stability (Dienes, 2014, 2016). Visualizing posterior probability as a function of prior plausibility helps reveal how much of the apparent strength of a claim depends on prior optimism.

**Figure 1:**
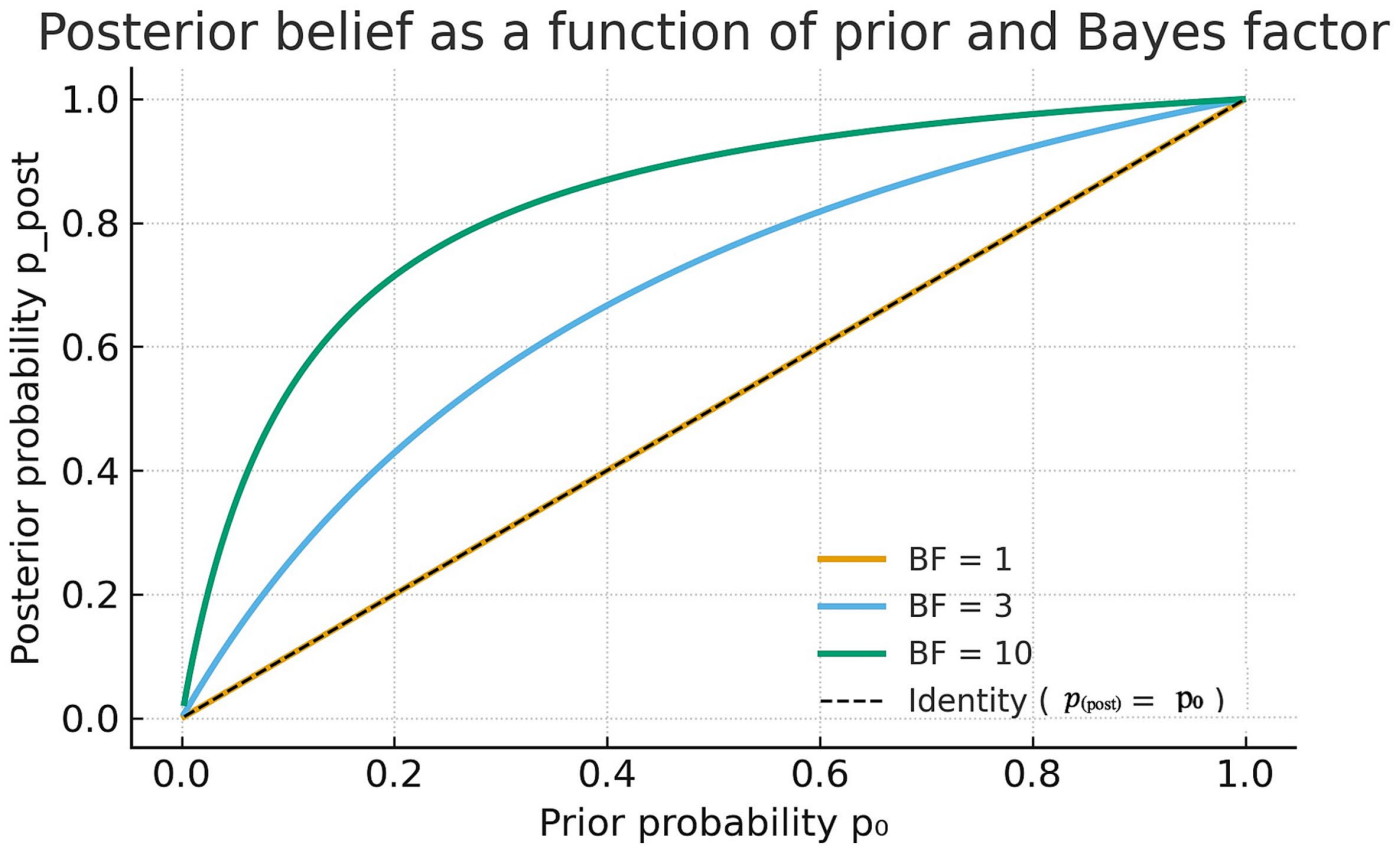
Posterior probability *p*_post_ as a function of prior probability *p*_0_ for three Bayes factors (*BF*_10_ = 1, 3, 10). The dashed diagonal line represents the identity (*p*_post_ = *p*_0_). Weak evidence (BF = 1) produces no change in belief; moderate evidence (*BF*_10_ = 3) yields a modest update; and strong evidence (*BF*_10_ = 10) sharply increases posterior probability for the same prior.

Figure 1 shows the posterior probability $p_{\text{post}}$ as a function of the prior probability $p_{0}$ for three representative Bayes factors ( $BF_{10}=1,3,10$ ). The curves illustrate how stronger evidence (larger Bayes factors) steepens the relationship between prior and posterior belief, while weak evidence ( BF≈1 ) leaves the posterior essentially unchanged from the prior. This visualization clarifies the logic of the Bayesian audit: the evidential impact of a study depends jointly on the data (through the Bayes factor) and on the prior plausibility of the claim.

### Step 6: Synthesize proportional evidence

The final step integrates the results into a qualitative synthesis. The goal is not to deliver a binary verdict of “true” or “false,” but to assess whether the strength of the claim’s language is proportional to the level of evidential support. A claim with posterior probability near 0.9 may justify confident language, while one near 0.3 warrants a tentative formulation. When applied systematically, such proportional judgments could realign scientific writing with the underlying uncertainty of inference (Jaynes, 2003; Wagenmakers et al., 2018).

The six steps together provide a transparent and reproducible protocol for connecting statistical outcomes to epistemic conclusions. In the following section, we apply this framework to a well-known psychological finding—the social priming of walking speed—to illustrate how a Bayesian audit can reveal the difference between rhetorical and evidential strength.

## Case study: social priming and walking speed

The “elderly priming” study of Bargh et al. (1996) has become a textbook example of how strong theoretical claims can arise from weak statistical evidence. The re-analysis presented below serves an illustrative purpose only, aimed at showing how the Bayesian audit framework can be applied to an existing finding. In the original experiment, students were exposed to scrambled-sentence tasks containing words associated with old age (such as “Florida,” “gray,” or “retired”). Participants in the priming condition were reported to walk more slowly when leaving the laboratory than those in a control condition. The authors interpreted this as evidence that unconscious activation of an age-related concept could influence overt motor behavior, illustrating automaticity in social behavior.

### Step 1: Identifying the claim

The central claim is causal and general: exposure to elderly-related words *causes* slower walking speed. It extends beyond the specific experimental conditions, implying that automatic activation of social stereotypes can directly alter physical behavior. The strength of this claim justifies scrutiny under a Bayesian audit because it invokes a complex causal chain (semantic activation → conceptual priming → motor behavior) that challenges prior psychological and neurobiological expectations.

### Step 2: Prior plausibility

Prior plausibility concerns how believable the claim is before any data are observed. Given the limited evidence for unconscious-to-motor causation at the time, the prior probability that such an effect is real can reasonably be considered low. Three priors can be defined for illustrative purposes:

-
$p_{0}=0.20$ , representing an optimistic prior (the effect is somewhat plausible);
-
$p_{0}=0.10$ , a moderate prior;
-
$p_{0}=0.05$ , a skeptical prior.

Prior odds are defined as

$$\mathrm{Odd}s_{\left(\text{prior}\right)}=\frac{\;p_{0}\;}{\left(1-p_{0}\right)}$$

For $p_{0}=0.10$ , prior odds equal 0.111 ; for $p_{0}=0.20$ , they equal 0.25.

### Step 3: Likelihood from the data

The original study reported t(28)≈2.0 , corresponding to p≈0.05 and an approximate standardized mean difference of d≈0.75 . Translating this result into a Bayes factor using the Jeffreys–Rouder approximation yields roughly

$$BF_{10}\approx 3$$

Indicating only *anecdotal-to-moderate* evidence in favor of the effect (Jeffreys, 1961; Wagenmakers et al., 2010; Dienes, 2014).

Because the original claim is directional, a one-sided Bayes factor could be considered more closely aligned with the hypothesis under scrutiny. Using a one-sided specification would moderately increase the evidential strength, without altering the qualitative conclusions of the audit.

Later replication attempts (Doyen et al., 2012; Harris et al., 2013) found Bayes factors close to 1, indicating limited evidential impact at the level of individual studies. However, in principle, evidence can be combined across studies using likelihood-based approaches, and the cumulative evidential impact depends on how such aggregation is performed.

### Step 4: Posterior updating

Posterior odds are obtained as the product of the prior odds and the Bayes factor:

$$\mathrm{Odd}s_{\left(\text{post}\right)}=BF_{10}\times \mathrm{Odd}s_{\left(\text{prior}\right)}$$

The posterior probability that the claim is true is then

$$p_{\left(\text{post}\right)}=\frac{\mathrm{Odd}s_{\left(\text{post}\right)}\;}{\left(1+\mathrm{Odd}s_{\left(\text{post}\right)}\right)}$$

The analysis shows that even under optimistic priors ( $p_{0}=0.20$ ), the posterior probability remains below 0.5, meaning that the evidence is insufficient to justify confident belief in the effect. For more skeptical priors ( $p_{0}\leq 0.10$ ), the posterior probability drops to 0.25 or less. For completeness, a neutral prior (*p*₀ = 0.50) can also be considered; under such an assumption, the resulting posterior remains moderate, reinforcing the qualitative conclusions of the audit.

### Step 5: Sensitivity and replication

Sensitivity analysis can be visualized by plotting $p_{\text{post}}$ as a function of prior probability for different Bayes factors. When $BF_{10}=3$ , the posterior curve rises slowly and remains below 0.5 unless *p*₀ exceeds 0.25. When replication attempts yield $BF_{10}\approx 1$ , the posterior remains unchanged at the level of individual studies. However, in principle, evidence can accumulate across studies, and the overall impact depends on how such evidence is combined.

### Step 6. Proportional synthesis

The audit concludes that the evidential support for the claim “elderly priming causes slower walking” is weak. The posterior probability remains low across plausible priors and collapses under replication. The magnitude of belief justified by the data is far below the certainty implied by the original wording. A proportional reformulation might be: *“There is weak and inconsistent evidence that exposure to elderly-related words may influence walking speed under certain conditions.”*

Such recalibration illustrates the broader purpose of a Bayesian audit: to bring scientific language into alignment with inferential logic. In doing so, it transforms the replication crisis from a failure of results into a lesson in epistemic humility.

## Discussion

The Bayesian audit applied to the social priming case highlights a broader point about the logic of scientific inference. A key distinction underlying this framework is between evidence and belief. Evidence, in the Bayesian sense, is a property of the data and is quantified by likelihood-based measures such as the Bayes factor. Belief, by contrast, reflects the integration of this evidence with prior assumptions. The Bayesian audit explicitly separates these two levels, aiming not to collapse them into a single quantity but to examine how they interact in the evaluation of scientific claims. Statistical results, however striking, do not speak for themselves; their meaning depends on the web of prior expectations and alternative explanations within which they are interpreted. When prior plausibility is low, even apparently significant findings may result in posterior beliefs that remain limited relative to the strength of the original claim, despite the evidential shift provided by the data. The audit therefore translates the replication crisis from a methodological problem into an epistemic one: not simply a shortage of replications, but a mismatch between how scientists *express belief* and how much belief the data actually warrant.

This mismatch is not unique to psychology. It reflects a general feature of scientific reasoning: the tendency to conflate evidence with persuasion. As Jaynes (2003) argued, probability theory provides a consistent extension of logic to uncertain reasoning; yet, in everyday scientific practice, claims often exceed what that logic allows. The Bayesian audit makes the logical structure explicit by decomposing each claim into priors, likelihoods, and updates, and by evaluating whether the resulting posterior belief justifies the strength of the conclusion. In this sense, the audit is not a statistical test but a *discipline of proportion*—a way to ensure that conclusions are commensurate with evidence.

The philosophical roots of this idea reach back to both Popper and de Finetti. Popper (1959) maintained that scientific statements must be falsifiable; de Finetti (1937) reframed probability as a measure of coherent belief. A Bayesian audit merges these perspectives by treating each claim as a provisional bet against reality. A strong claim with weak evidence is a risky bet; a cautious claim with solid evidence is a rational one. Through this lens, replication failures are not crises but recalibrations: instances in which accumulated data revise our odds toward coherence [see also recent discussions of belief updating in the context of replication, e.g., (Korbmacher et al., 2023)].

Methodologically, the audit complements existing Bayesian tools such as Bayes factors and reverse-Bayes analyses (Held and Ott, 2018; Dienes, 2014, 2016). Its novelty lies in shifting the focus from isolated results to the *claims themselves*. By framing empirical assertions as hypotheses subject to probabilistic evaluation, the audit invites a more transparent dialogue between data, theory, and rhetoric. A field that routinely subjects its conclusions to such audits would not only reduce false discoveries but also promote conceptual clarity—an outcome as important as replication itself.

In the case of social priming, the audit makes explicit what the replication record already implies: the evidential support is limited relative to the strength of the original claim, the posterior belief low, and the language should reflect that proportionality. More generally, it demonstrates that Bayesian reasoning can provide not only a method for statistical analysis but also a logic for scientific humility. When applied systematically, Bayesian audits could help turn reproducibility from a defensive exercise into a constructive process of epistemic self-correction—one in which every claim is both a finding and a question about how much we should believe it.

A limitation of the Bayesian audit is that its conclusions depend on subjective elements, particularly the specification of prior plausibility. While this subjectivity is an inherent feature of Bayesian reasoning, it also represents a source of variability across analysts. The audit therefore does not eliminate subjectivity, but makes it explicit and open to scrutiny.

It is also important to note that the evidential value of measures such as Bayes factors depends on the quality of the underlying data. Low-powered designs or biased sampling may limit the reliability of any inferential measure, as the evidential value of likelihood-based quantities ultimately depends on the quality of the underlying data (Witte and Zenker, 2017; Krefeld-Schwalb et al., 2018). In the present case, the low statistical power of the original study provides independent grounds for skepticism, as it implies that only very large effects could have been reliably detected. This observation reinforces the conclusions of the Bayesian audit, highlighting that the apparent evidential support is fragile when considered in light of the study’s design.

The Bayesian audit operates on reported empirical results, treating them as inputs to inferential updating. It does not attempt to evaluate or reconstruct the design quality of the underlying studies. However, this does not imply that the evidential outputs of the audit are independent of data quality. On the contrary, the strength of the inferred evidence must always be interpreted in light of how the data were generated. In this sense, the audit does not replace considerations of study quality, but assumes them as a background condition that constrains the interpretation of evidential measures.

In this perspective, the Bayesian audit should be understood as a tool for interpreting evidential strength, not for establishing it independently of the conditions under which data are produced.

## Applications

Although illustrated here using a case study from social psychology, the Bayesian audit can be applied across a wide range of empirical domains where the proportionality between claim and evidence is critical.

- Neuroscience: In neuroimaging research, Bayesian audits can be used to assess whether claims about brain–behavior relationships are commensurate with the strength of the underlying statistical evidence. For instance, a reported activation pattern could be re-evaluated in terms of posterior belief rather than thresholded significance, helping to calibrate interpretive claims in fMRI or PET studies.
- Clinical trials: In medical and clinical research, the audit can complement traditional Bayesian analyses by quantifying whether conclusions about treatment efficacy are proportionate to the data, given prior biological plausibility and existing evidence. This is particularly useful in early-phase or low-prevalence studies, where small effect sizes can otherwise be overstated.
- Meta-analyses: At the meta-analytic level, Bayesian audits can evaluate the coherence of aggregated evidence with the strength of general conclusions drawn in the literature. By combining prior expectations with observed heterogeneity and Bayes factors from individual studies, one can assess whether the synthesized claims remain epistemically justified.

More generally, the audit can serve as a transparent reporting tool for any empirical field where strong claims are based on uncertain or indirect evidence—from cognitive modeling to AI benchmarking and policy evaluation. By making the logic of belief updating explicit, it offers a unifying framework for proportional reasoning across scientific disciplines.

## Conclusion

A Bayesian audit provides a transparent and reproducible way to evaluate whether scientific claims are proportional to the evidence that supports them. By making priors explicit, quantifying the strength of the likelihood, and updating to posterior belief, the audit exposes the epistemic distance between statistical detection and rational conviction. The case of social priming illustrates how this distance can be large: a small experimental effect, amplified by narrative certainty, produced a claim that far exceeded its evidential basis. Reframing such findings through the logic of probability does not weaken science—it strengthens it. The audit thus extends Bayesian inference from data analysis to claim evaluation, promoting a culture of proportional reasoning and linguistic precision. In the long run, this alignment between rhetoric and evidence may be as crucial to scientific progress as replication itself.
